# Solid Lipid Nanoparticles Delivering a DNA Vaccine Encoding *Helicobacter pylori* Urease A Subunit: Immune Analyses before and after a Mouse Model of Infection

**DOI:** 10.3390/ijms25021076

**Published:** 2024-01-16

**Authors:** Jasmine E. Francis, Ivana Skakic, Debolina Majumdar, Aya C. Taki, Ravi Shukla, Anna Walduck, Peter M. Smooker

**Affiliations:** 1 School of Science, RMIT University, 264 Plenty Road, Bundoora, VIC 3083, Australia; j.ericafrancis@gmail.com (J.E.F.); ivana.skakic@rmit.edu.au (I.S.); debolina.majumdar@csiro.au (D.M.); ravi.shukla@rmit.edu.au (R.S.); anwalduck@csu.edu.au (A.W.); 2 Melbourne Veterinary School, Faculty of Science, The University of Melbourne, Parkville, VIC 3010, Australia; aya.taki@unimelb.edu.au

**Keywords:** *Helicobacter pylori*, DNA vaccine, solid lipid nanoparticles, urease alpha subunit

## Abstract

In this study, novel solid lipid particles containing the adjuvant lipid monophosphoryl lipid A (termed ‘SLN-A’) were synthesised. The SLN-A particles were able to efficiently bind and form complexes with a DNA vaccine encoding the urease alpha subunit of *Helicobacter pylori*. The resultant nanoparticles were termed lipoplex-A. In a mouse model of *H. pylori* infection, the lipoplex-A nanoparticles were used to immunise mice, and the resultant immune responses were analysed. It was found that the lipoplex-A vaccine was able to induce high levels of antigen-specific antibodies and an influx of gastric CD4^+^ T cells in vaccinated mice. In particular, a prime with lipoplex-A and a boost with soluble UreA protein induced significantly high levels of the IgG1 antibody, whereas two doses of lipoplex-A induced high levels of the IgG2c antibody. In this study, lipoplex-A vaccination did not lead to a significant reduction in *H. pylori* colonisation in a challenge model; however, these results point to the utility of the system for delivering DNA vaccine-encoded antigens to induce immune responses and suggest the ability to tailor those responses.

## 1. Introduction

*Helicobacter pylori* has been classified as a class I carcinogen by the World Health Organisation (WHO) since 1994 [1]. Although it is believed to colonise almost 50% of the global population, most infected individuals remain asymptomatic. The pathogen utilises several mechanisms to evade host immune responses and establish suitable environmental conditions for its survival and colonisation within the gut. The urease enzyme of *H. pylori* is a key virulence factor that facilitates colonisation of the pathogen in the gut. Comprising subunits A and B, urease was one of the first bacterial antigenic components identified for vaccinations against *H. pylori* [2]. *H. pylori* urease secretion results in increased pH levels, thus producing an environment more favourable for survival against gastric acid and pathogen persistence [3]. A recombinant multimeric vaccine utilising *H. pylori* urease demonstrated induction of antigen-specific CD4+ T cells in immunised Mongolian gerbils [4]. The UreA subunit has a crucial role in the activation of urease due to its interaction with Heat shock protein 60 (Hsp60), a highly abundant protein of *H. pylori* which acts as a molecular chaperone to protect other proteins against heat-induced aggregation [5]. The adoptive transfer of UreA-specific T cells was found to confer a threefold reduction in gastric bacterial loads [6], and more recently oral vaccination utilising UreA demonstrated protective responses to *H. pylori* challenges [7]. Despite substantial global efforts, *H. pylori* remains without a licenced vaccine [8] and therefore is a target for novel vaccine strategies including the use of nanomaterials [9,10].

Almost thirty years ago it was discovered that intradermal injection of DNA could generate immune responses to encoded antigens in mice [11]. The original concept of gene delivery using plasmid vectors was to create a shuttle platform for the delivery of a gene to live mammalian cells for expression, for gene therapy purposes [12]. It was soon discovered that this platform had functionality beyond gene delivery, as encoded proteins could stimulate both humoral and cellular immunity and offered intrinsic stimulatory properties beyond that of protein-based vaccines [13]. In the decades since this observation, DNA vaccines have become commercialised, and several are available on the veterinary market, including an equine West Nile virus vaccine [14] and a canine melanoma vaccine [15], and a DNA vaccine encoding the spike protein of SARS-CoV-2 was tested in animals and humans and subsequently rolled out into the Indian market for emergency use in 2022 [16,17]. This demonstrated proof of principle in the human clinical setting.

A considerable advantage of DNA vaccination, similar to mRNA vaccines, is that antigens expressed in vivo are readily presented in complex with both MHC class I and II, allowing the activation of CD4^+^ and CD8^+^ T cells for either humoral or cellular immune responses, which is a feature lacking in many non-live vaccine platforms [18]. Despite these advantages, the major barrier to the widespread clinical application of DNA vaccines is their limited efficacy in humans to date (notwithstanding the excellent result against SARS-CoV-2 mentioned above). Poor cellular uptake of naked plasmid DNA remains a major challenge to transfection in vivo, resulting in insufficient immune responses and a lack of protective efficacy. Investigation into the fate of injected DNA found that 99% of DNA injected in the muscle and skin is degraded by endonucleases in the interfibrillar space [19]. This significant challenge must be overcome before DNA vaccines can experience broad clinical application in humans. One way to accomplish this is to employ needle-free vaccination using a PharmaJet Tropis device—this is how the Indian DNA vaccine is delivered [16]. In an alternative approach to improve the efficacy of DNA vaccines, linking DNA to a nanoparticle carrier has been proposed as a method to improve vaccine uptake and immune priming [20]. Particle-based carrier systems can protect DNA from endonucleases and increase immune cells’ antigen uptake [21,22]. Solid lipid nanoparticles can bind or load molecular cargo via electrostatic interaction or encapsulation. Lipids are generally well tolerated and, in some cases, metabolised by cells for growth. Fast cellular uptake is desirable as it increases the uptake and processing of antigens in vivo, allowing a faster immune response.

Cationic solid lipid nanoparticles (SLN) with increasing amounts of lipid adjuvant, monophosphoryl-lipid A (MPLA), have been demonstrated to be stable and non-cytotoxic nanoparticles which act as vaccine adjuvants by activating human macrophage cells in vitro. MPLA is an attenuated analogue of lipopolysaccharide (LPS) and a potent TLR4 ligand, making it an ideal vaccine antigen and suitable lipid species for incorporation into SLN formulations [23]. It has been studied extensively as an immunomodulatory agent and vaccine adjuvant and is now an FDA approved adjuvant which is used in the human HPV vaccine Cervarix [24]. We have previously demonstrated that a plasmid vector encoding *Helicobacter pylori* candidate antigen urease subunit A, pcDNA-UreA, was incorporated into lipoplexes synthesised from solid lipid nanoparticles (MPLA nanoparticles) [25]. The resultant lipoplex-A particle complexes were characterised and, importantly, found to strongly bind and protect plasmid DNA, demonstrating functionality as a potential DNA vaccine nanocarrier platform. Lipoplexes were able to successfully transfect murine dendritic cells in vitro and were found to be non-cytotoxic and readily internalised by cells. Here we take these lipoplexes and test their immunogenicity in vivo in a murine model of *H. pylori* infection. In addition, we study the results of vaccination with DNA alone and vaccination with a DNA priming dose and a protein boost, analysing humoral responses before and after challenge with *H. pylori* and cellular responses after. 

## 2. Results

### 2.1. Synthesis of Lipoplex-A Vaccine and Control Vaccine

**Figure 1:**
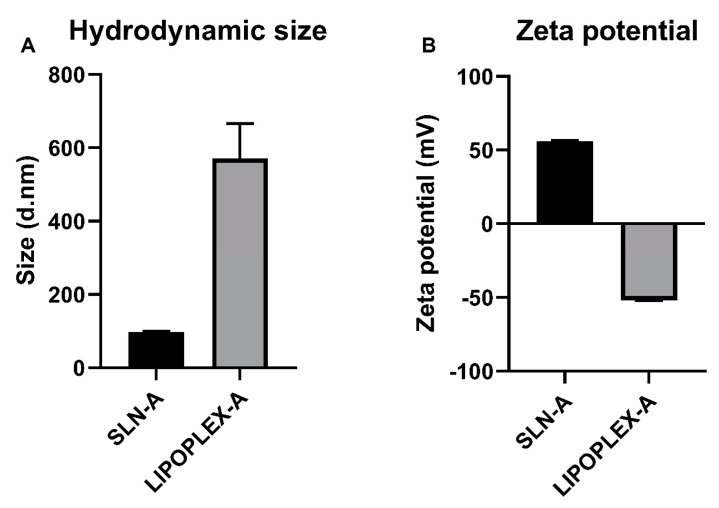
The size (**A**) and zeta potential (**B**) of SLN-A and lipoplex-A formulations were analysed via DLS. Lipoplexes were synthesised at a 1:2 ratio (*w*/*w*) DNA:SLN. SLN-A particles had an average diameter of 98.0 (±6.9) nm with an average zeta potential of 55.9 (±3.7) mV. Lipoplex-A were found to have a greater diameter of 571.4 (±94.9) nm and a zeta potential of −51.9 (±2.3) mV.

The synthesis of the solid lipid nanoparticles and the construction of the lipoplexes containing the UreA-encoding DNA vaccine was undertaken as described by Francis et al. (2020) and Francis et al. (2022) [25,26]. The size and zeta potential of SLN-A and lipoplex-A prepared for use as a vaccine formulation were analysed via DLS. Size measurements indicated that SLN-A(H) particles had an average diameter of 98.0 (±6.9) nm with an average zeta potential of 55.9 (±3.7) mV (Figure 1). Lipoplex-A samples were found to have a diameter of 571.4 (±94.9) nm and a zeta potential of −51.9 (±2.3) mV. 

### 2.2. TEM Characterisation of SLN-A and Lipoplex-A for Vaccine Preparation

Adjuvanted SLN-A nanoparticles were used to formulate the lipoplex-A vaccine. 

**Figure 2:**
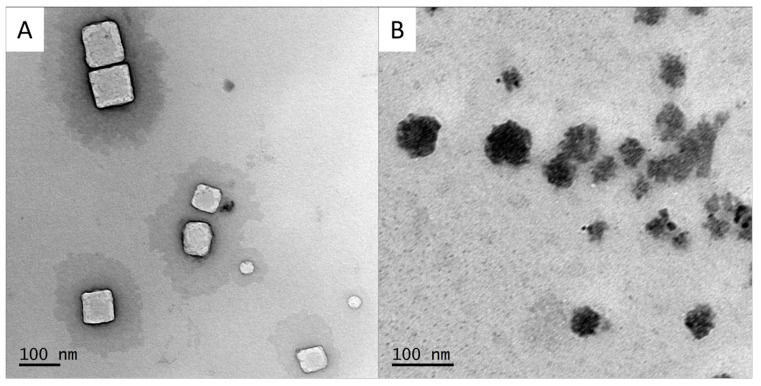
TEM micrograph of (**A**) SLN-A and (**B**) lipoplex-A used for vaccination of mice. SLN-A were found to be heterogenous in size and shape with an average diameter 78.1 ± 41.3 nm. Lipoplex-A had an average size of 70.2 ± 43.0 nm and were also heterogenous in size and shape, with undefined edges due to coating with DNA.

SLN-A particles shown in Figure 2A were found to be heterogenous in size and shape, appearing as a mixture of spherical and cuboid-shaped particles. The average particle diameter was found to be 78.1 ± 41.3 nm. 

Lipoplex-A particles were found to have an average diameter of 70.2 ± 43.0 nm (Figure 2B). Particles appear more heterogenous in size and shape than SLN-A, with rough, undefined edges. Particle sizes were measured more accurately by TEM, thus are significantly smaller than DLS measurements of particles in solution, which are prone to aggregation. 

### 2.3. Humoral Responses to Vaccination and Challenge

#### 2.3.1. Pre-Challenge Antigen-Specific IgG, IgG1, and IgG2c Antibody Levels

**Figure 3:**
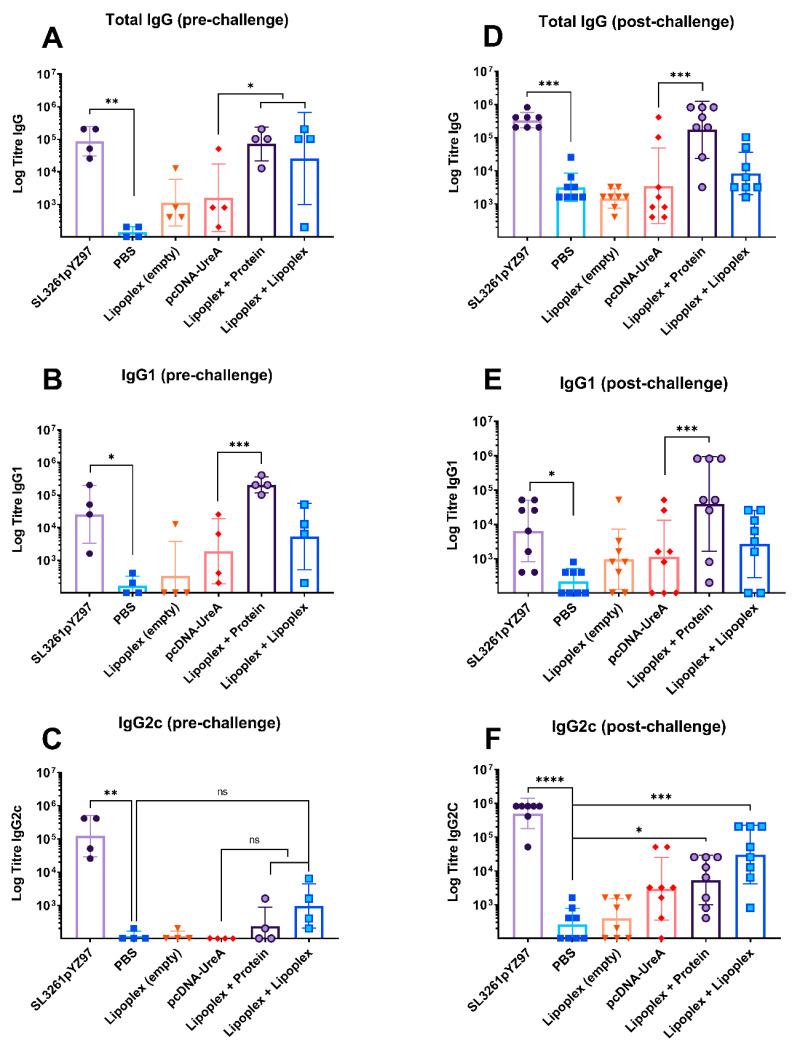
Antibody titres before (**A**–**C**) and after (**D**–**F**) *H. pylori* challenge. Mice were vaccinated as described in table in Section 4.2. Titres of total IgG, IgG1, and IgG2c isotypes were determined 128 days after the first vaccination (pre-challenge) and 21 days after *H. pylori* challenge (post-challenge). The bars represent the mean of 4 mice per group (pre-challenge) and 8 mice per group (post-challenge), ±SEM. * *p* ≤ 0.05, ** *p* ≤ 0.01, *** *p* ≤ 0.001, **** *p* ≤ 0.0001. Non-significant differences are denoted ns.

Serum analysis of whole IgG from individual mice prior to *H. pylori* challenge (‘pre-challenge’) is shown in Figure 3A and indicates humoral responses generated by vaccination. Mouse sera assayed for whole IgG showed significant levels of IgG in the SL3261pYZ97 control sera which had a mean titre of 121,600, which was significantly higher than the sham immunized PBS control which were challenged with *H. pylori* (*p* = 0.007). The lipoplex-A with protein boost group and lipoplex-A boost group also had significantly higher titres than the naked DNA control (*p* = 0.03), with mean titres of 105,600 and 102,450, respectively. This demonstrates that the lipoplex-A vaccine induced strong humoral responses.

Subtype IgG1 and IgG2c responses were measured to indicate Th1/Th2 bias in antibody responses. Mouse sera assayed for IgG1 subtype antibodies showed significant levels of IgG1 in the SL3261pYZ97 control sera compared to the PBS control (*p* = 0.03), with a mean titre of 70,800. The lipoplex-A with protein boost group had significantly higher titres compared to naked DNA (*p* = 0.001) (Figure 3B) with a mean titre of 230,400, indicating a strong Th2 response to the protein boost vaccine. 

Mouse sera assayed for IgG2c as an indicator of Th1 responses (Figure 3C) showed significant levels of IgG2c in the SL3261pYZ97 control sera (*p* = 0.008) with a mean titre of 224,000. Neither the naked DNA nor the lipoplex-A groups had significant titres of IgG2c.

#### 2.3.2. Endpoint Antigen-Specific IgG, IgG1, and IgG2c Antibody Levels

Significant levels of UreA-specific IgG were also detected in groups after the *H. pylori* challenge, indicating strong humoral responses. Mouse sera assayed for endpoint IgG titres showed significant levels of IgG in the SL3261pYZ97 positive control sera (*p* = 0.0002), as well as the lipoplex-A with protein boost group (*p* = 0.0002) (Figure 3D), which had mean titres of 380,400 and 413,200, respectively. It is notable that the *H. pylori* challenge alone (PBS control group) stimulated a small increase in total IgG by 21 days post challenge (Figure 3D), but that increases in IgG1 and IgG2c were minimal (Figure 3E,F).

Endpoint titres of the IgG1 antibody shown in Figure 3E showed significant antibody levels in sera from mice which received the SL3261pYZ97 control, which had a mean titre of 20,300––significantly increased compared to PBS alone (*p* = 0.02). Notably, mice in the lipoplex-A vaccine with protein boost group had the highest IgG1 titre of all groups (323,300), which was significantly higher than those generated by naked DNA (*p* = 0.0002).

Mouse sera assayed for the IgG2c subtype shown in Figure 3F showed significant levels of IgG2c in the SL3261pYZ97 control sera (*p* ≤ 0.0001), the lipoplex-A with protein boost group (*p* = 0.03), and the lipoplex-A with lipoplex-A boost group (*p* = 0.0008) compared to the PBS control, with mean titres of 650,900, 12,350 and 88,900, respectively. The difference in IgG2c levels between the lipoplex-A vaccine groups compared to the naked DNA group was not significant.

Both experimental lipoplex-A groups generated significant levels of whole IgG following the *H. pylori* challenge, with the heterologous protein boost group generating the strongest UreA-specific whole IgG response and also the strongest IgG1 response. This is indicative of a bias towards a Th2 response induced by the heterologous prime-boost regime in mice vaccinated with lipoplex-A and boosted with recombinant UreA protein, while a homologous prime-boost with lipoplex-A induced a Th1 response as indicated by elevated IgG2c titres. 

### 2.4. Analysis of Gastric Infiltrates

**Figure 4:**
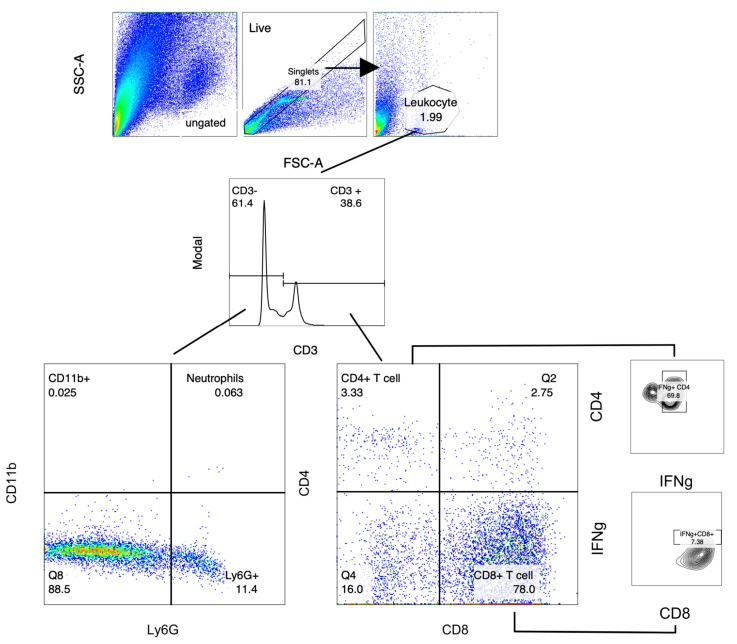
Gating strategy used for analysis of lymphocyte populations represented by a pseudo-colour density plot. Single cells were gated according to their forward scatter (FSC-H and FSC-A) characteristics to exclude aggregates and define a leukocyte gate. Leukocytes were subdivided into CD3^+^ CD4^+^ T cells, IFNγ^+^ CD4^+^ T cells, and CD8^+^ T cells and IFNγ^+^ CD8^+^ T cells. CD3^−^ cells’ macrophages were defined as (CD11b^+^) and their neutrophils as (CD11b^+^/Ly6G^+^).

Local lymphocyte populations in the stomachs of mice were characterised 21 days post challenge. Leukocyte populations in mouse stomach tissue were stained for relevant surface markers and analysed via flow cytometry (Figure 4). Statistical significance was determined using the Kruskal–Wallis test with Dunn’s multiple comparisons test, and significance is represented as * *p* ≤ 0.05, ** *p* ≤ 0.01, *** *p* ≤ 0.001. For each experiment, cells were isolated from *n* = 7–8 individuals per group. Cell populations were differentiated via staining surface markers (CD3, CD4, CD8, Ly6G, and CD11b) and intracellular staining for IFNγ^+^ as described above. Lymphocytes were subdivided into CD3^+^CD4^+^ T cells, IFNγ^+^ CD4^+^ T cells, CD8^+^ T cells, and IFNγ^+^ CD8^+^ T cells. CD3 negative cells were defined as macrophages (CD11b^+^) and neutrophils (CD11b^+^/Ly6G^+^). A minimum of 10,000 cells per sample were collected for analysis using FlowJo software version 8. Absolute cell counts were determined via backgating to cell counts through the live gate.

### 2.5. Analysis of Immune Cell Populations from Mouse Stomach Tissue

**Figure 5:**
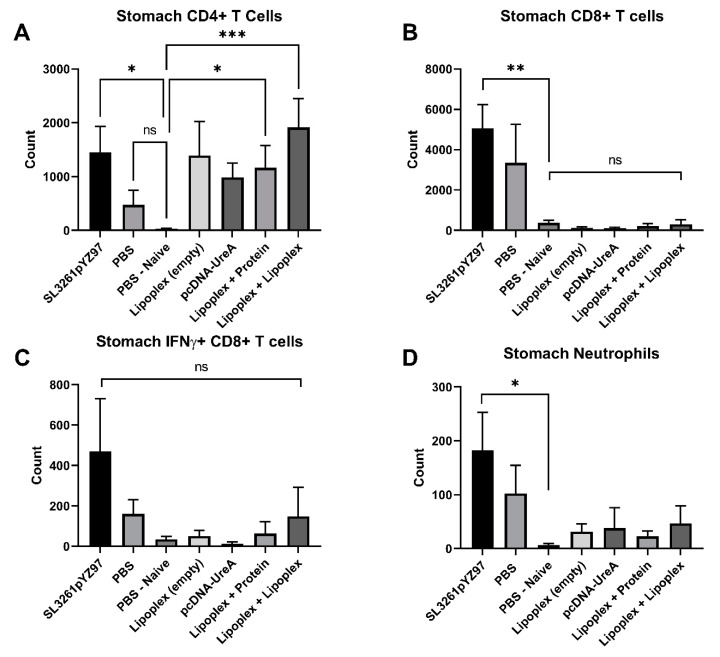
Mean counts of infiltrating immune cells isolated from stomachs of mice 21 days after challenge with *H. pylori*. **A** (CD4^+^), **B** (CD8^+^), **C** (IFNγ^+^ CD8^+)^, **D** (Neutrophils). The lipoplex vaccines induced infiltrates of CD4^+^ T cells, but minimal levels of CD8^+^ or neutrophils. Data represent the mean counts from 7–8 mice per group, ±SEM. * *p* ≤ 0.05, ** *p* ≤ 0.01, *** *p* ≤ 0.001. Non-significant differences are denoted ns.

Analysis of gastric infiltrating T cells in the stomach at 21 days after *H. pylori* challenge is shown in Figure 5. Both lipoplex-A vaccine boosted with protein (*p* = 0.01) and lipoplex-A (*p* = 0.0007) induced significant levels of CD4^+^ T cells compared to PBS naïve mice, as did the SL3261pYZ97 control (*p* = 0.01), as shown in Figure 5A. Figure 5B shows the populations of gastric infiltrating CD8^+^ T cells in vaccinated mice. The SL3261pYZ97 control vaccine induced significant levels of CD8^+^ T cells compared to PBS naïve mice (*p* = 0.0075); however, very low levels of CD8^+^ T cells were detected in lipoplex-A and control groups. No significant difference was detected in IFNγ^+^ CD8^+^ T cell populations between groups. Only the SL3261pYZ97 control vaccine induced significant levels of neutrophils compared to PBS naïve mice (*p* = 0.03). 

### 2.6. Determination of H. pylori Burdens from Mouse Stomachs

**Figure 6:**
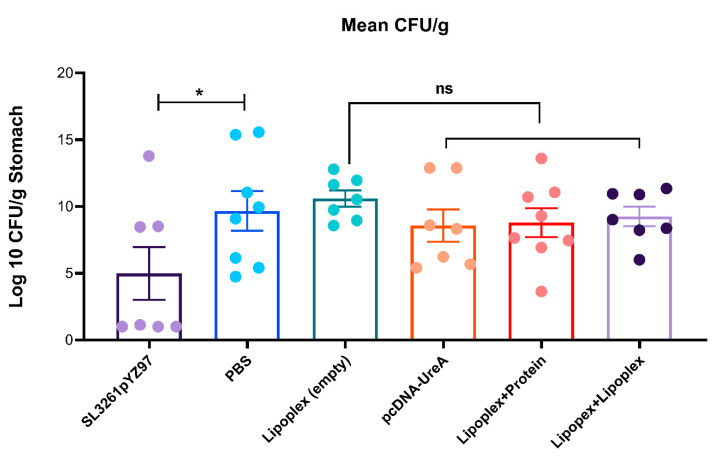
qPCR detection of mouse stomach burdens of *H. pylori* in vaccinated mice. Mice vaccinated with SL3261pYZ97 control vaccine had significantly reduced levels of gastric *H. pylori* compared to the infected control (* *p* = 0.03, one-tailed). No significant difference was detected between mice vaccinated with empty vector control or lipoplex-A vaccine. Data represent the analysis of 7–8 mice per group, ±SEM. Non-significant differences are denoted ns.

As the model we have used is a challenge model, the bacterial burdens were assessed. Levels of gastric *H. pylori* were detected in mouse stomach homogenate via qPCR of *H. pylori* 16S DNA. Stomach DNA samples were analysed via qPCR to detect the presence of *H. pylori* 16S rDNA and quantify burdens against a set of known *H. pylori* standards ranging from 0.0001–100 ng, as previously described [27]. Samples were analysed via real-time qPCR and underwent 30 cycles of amplification with a melting temperature of 56 °C, using group C (naïve control) to set the baseline above which a signal threshold was applied. Bacterial counts per gram of stomach tissue were determined via extrapolation from the standard curve of known *H. pylori* DNA concentrations. Results are shown in Figure 6. Statistical significance was determined using the Mann–Whitney test with Dunn’s Multiple Comparisons test and significance is represented as * *p* ≤ 0.05.

Mice vaccinated with SL3261pYZ97 control vaccine had a 1.9-fold reduction in *H. pylori* colonization compared to the infected control (*p* = 0.03, one-tailed). Mice vaccinated with lipoplex-UreA vaccine did not have reduced levels of colonisation compared to the infection control (PBS) or empty lipoplex groups. 

## 3. Discussion

In this study, adjuvanted solid lipid nanoparticles loaded with a DNA vaccine against *H. pylori* were developed (lipoplex-A), characterised, and tested for the ability to induce humoral immune responses in mice. Humoral and cellular responses after a live *H. pylori* challenge were also assessed, as was the protective efficacy (none was seen). In this study the lipoplex DNA vaccine was compared to a live attenuated *Salmonella enterica* serovar Typhimurium (*S. typhimurium* SL3261pYZ97), expressing UreA and UreB subunits as a positive control, as this has previously been shown to confer a 1–2 log reduction in *H. pylori* colonisation [28,29]. No *Salmonella* vector vaccines have thus far been approved for use in humans. A recombinant *S. enterica* Typhimurium strain expressing *H*. *pylori* UreA has been tested in Phase 1 trials in human volunteers; however, it was poorly protective [30]. 

In this study, IgG antibody responses were investigated as the best indication of a humoral immune response. While mucosal immune responses to *H. pylori* are relevant to protection, mouse infection models of *H. pylori* Sydney strain 1 (SS1) typically do not result in the generation of detectable mucosal IgA antibody responses [31]. Furthermore, it is understood that antibody responses do not play a role in protection against *H. pylori* [32]. The investigation of antibody responses undertaken here contributes to understanding the immunogenicity of the lipoplex-A vaccine, and to understanding the immunogenicity of nanoparticle-mediated DNA vaccines. This is a “platform” technology which will be applicable to a variety of antigen/pathogen combinations.

Characterisation of antibody levels in vaccinated mice revealed that both before and after the *H. pylori* challenge, mice vaccinated with a heterologous prime-boost (DNA followed by protein) trended towards a Th2-biased response with significantly higher IgG1 levels, while a homologous prime-boost with lipoplex-A generated a stronger IgG2c response and therefore indicated a Th1-biased immune response. These data suggest that a heterologous prime-boost regime where mice are primed with lipoplex-A and boosted with protein generates the strongest humoral response, while mice receiving no recombinant protein trended towards a cellular immune response. In future applications, this should allow the tailoring of the vaccination regime to the protective polarisation of the immune response.

Notably, both MPLA and CpG adjuvants have a known Th1 bias [33,34,35]. CpG is a known TLR9 agonist, favouring inflammation and Th1 differentiation [23]. Additionally, MPLA induces TLR4-mediated activation of antigen-presenting and innate immune cells which leads to Th1 differentiation and the induction of Th1-associated humoral responses [34,36]. Thus, the mice in group 7 which received two doses of lipoplex-A (Group G) had twice the exposure to both CpG and MPLA, both Th1-biased adjuvants, which likely drove this observed Th1-biased response, while mice in group 6 received only one dose of adjuvanted vaccine, followed by boosting with soluble UreA protein.

In previous studies, heterologous prime-boost vaccination regimes have been shown to produce a greater breadth and intensity of immune responses [37,38]. The use of a DNA prime followed by protein boost in previous studies has been shown to similarly augment Th2-biased immune responses [39]. Priming with DNA is effective at inducing strong antigen-specific T cell responses [40]. The mechanism behind the DNA prime-boost strategy is that in the initial vaccination, using DNA primes antigen-specific T cells by transfecting APCs for fast and effective MHC I and II presentation, allowing for an accelerated T cell response to a protein, toxoid, or vector boost [18]. These results indicate that the prime-boost regime influences vaccine outcomes for lipoplex-A and has implications for the design of future lipoplex vaccine studies. This prime-boost effect is notably important for considerations for future studies in the use of lipoplex-A against viruses or other pathogens which can be protected against using neutralizing antibodies.

In this study C57BL/6 mice were used as the *H. pylori* mouse infection model, which is well established in this strain [41,42]. While C57BL/6 mice are widely accepted as a suitable strain of mice to use in vaccine studies, they are known to generate Th1 dominant immune responses [43,44], which should be considered in the evaluation of vaccine efficacy in trials utilising C57BL/6 mice. It is notable that in the experiment described here, priming with a lipoplex-A vaccine and boosting with protein induced significant levels of IgG1 (Figure 3), which is indicative of a Th2 response. It would be interesting to undertake vaccination in BALB/c mice, which are predisposed to Th2 responses. It may be expected that after boosting with a soluble protein that the IgG1 level would be higher than we observed here in C56BL/6 mice. Many studies have utilised BALB/c mice in *H. pylori* vaccine studies; notably, both C57BL/6 and BALB/c wild-type strains are readily colonised using SS1 and are therefore suitable for this form of experiment [45].

Vaccine outcomes are heavily influenced by the route of administration [46,47,48]. In this study, we chose the subcutaneous route because a previous study [49] demonstrated that subcutaneous vaccination resulted in better immunological outcomes for DNA vaccination compared to intramuscular delivery, although other studies have reported strong immune responses from intramuscular DNA vaccination [50]. In addition, some studies suggest that intranasal and oral delivery may induce more effective immune responses against *H. pylori* due to the production of earlier systemic and gastric immune responses [51,52]. It is noteworthy that despite the subcutaneous delivery, significant mucosal CD4^+^ T cell responses were detected in our study, which indicates that the lipoplex-A vaccine has strong potential for use in vaccines directed against mucosal pathogens. In the recent application of a DNA vaccine encoding the SARS-CoV-2 spike protein, needle-free injection was used for delivery intradermally [16]. These parameters should be considered in future studies using the lipoplex-A vaccine model. 

Analysis of the response to the control vaccines in this study gave insight into the immunogenicity of the lipoplex-A particle system, as our results show that the empty vector control generated higher IgG and IgG1 titres than the PBS control. This effect is likely driven by a stimulatory effect of CpG motifs in the DNA vector and is an effect previously observed in mice vaccinated subcutaneously with empty DNA vector control vaccines [53]. 

Both experimental lipoplex-A-vaccinated groups generated significant antibody titres, with the heterologous protein boost group generating the strongest antibody response. These data should be used to inform future vaccine strategies using the lipoplex-A vaccine model. Previous studies have demonstrated the role of local gastric infiltrating CD4^+^ T cells in protection against *H. pylori* [29,54,55]. Our results overall indicate that the lipoplex vaccine has the potential to generate strong local CD4^+^ T cell responses, indicative of a cellular-mediated immune response. Previous studies using the SL3261PYZ97 vaccine have shown that the response to vaccination is limited to the site of infection in the stomach and was reflected in infiltrations of CD4^+^ T cells, macrophages, and neutrophils [29]. In our study, analysis of gastric infiltrating immune cells in the stomach found significant CD4^+^ T cell populations in the stomach tissue of both lipoplex-A vaccine groups, with the heterologous lipoplex-A vaccine group showing the strongest CD4^+^ response, other than the SL3261pYZ97 control. 

In terms of CD8^+^ T cell infiltration, the SL3261pYZ97 control vaccine induced the highest population of CD8+ T cells in the stomach, while negligible levels of CD8^+^ T cells were detected in the lipoplex- and DNA-vaccinated groups. Further investigation of IFNγ^+^ subsets of CD8^+^ T cells found no significant difference in IFNγ^+^ CD8^+^ T cell populations between groups; likewise, no significant difference in neutrophil populations was detected in vaccinated mice. Given that the attenuated Salmonella SL3261PYZ97 vaccine is known to be taken up in the Peyer’s patches of this intestine, and is able to replicate, if poorly, due to its attenuation, it is not surprising that this vaccination strategy was more effective at eliciting mucosal CD8+ T cell responses than subcutaneous administration of nanoparticles. 

Our results suggest that lipoplex-A administered subcutaneously is effective at promoting gastric infiltrating CD4^+^ T cells, but not granulocytes (macrophages and neutrophils) in local mucosal (stomach) tissues. The levels of CD8^+^ T cells detected were unexpectedly low (lower even than in the control mice exposed to *H. pylori* alone). This may be due to a reduced capacity of subcutaneously administered lipoplex-A to induce mucosal CD8^+^ cells, or possibly due to delayed responses compared to the live attenuated vaccine. We are therefore unable to draw conclusions about the efficacy of the lipoplex-A vaccine in stimulating mucosal CD8^+^ T cell responses at this stage. Further investigation into the cytokine responses of restimulated immune cells would yield useful information about the type of T helper response in vaccinated mice and the effect of augmenting the immune response through the prime-boost regime. 

The induction of antibody titres and CD4^+^ cell populations triggered by the lipoplex-A plus protein boost was suggestive that the vaccine would lead to reductions in *H. pylori* colonisation. However, in this experiment this was clearly not the case. Our study has, however, demonstrated that the lipoplex-A vaccine was as effective as a live attenuated vaccine at inducing strong humoral immune responses to the encoded UreA protein in immunised mice. The results also demonstrated that the prime-boost regime strongly augmented immune response to the lipoplex-A vaccine. A heterologous prime boost of lipoplex-A followed by protein was effective at generating significant levels of the IgG1 subtype antibody, indicative of a Th2-mediated response, while a homologous prime-boost regime of lipoplex-A generated high levels of IgG2c antibodies, indicative of a Th1-mediated response. 

Subcutaneously administered Lipoplex-A was found to induce CD4^+^ T cells that are home to the gastric mucosa, at levels that were significantly higher than the negative control, and similar to an orally administered live attenuated vaccine. While the lipoplex-A vaccine was not found to be protective against a *H. pylori* challenge, these results demonstrate that the lipoplex-A model has the potential to be an effective vaccine delivery system and should be further investigated using different routes of administration for its efficacy against other pathogens. In this context, we note that in a previous publication we have demonstrated that the challenge model used here was able to demonstrate significantly reduced colonization of mice after vaccination with a different type of nanoparticle [27]. The comparison between the two vaccines may be very illustrative in the design of future vaccines against *H. pylori.*

## 4. Materials and Methods

All animal experiments were undertaken with approval from the RMIT Animal Ethics Committee under the Animal Ethics Committee approval number AEC1820. All experiments and procedures were undertaken following approved animal handling and experimental protocols. 

### 4.1. Formulation of Vaccines and Characterisation by Dynamic Light Scattering (DLS) and TEM

The formulation of the lipoplexes and their characterisation were as previously described [25,26,56]. Briefly, SLNs were synthesized including the adjuvant lipid MPLA from *Salmonella enterica* serotype Minnesota Re 595 (Sigma, Clayton, VIC, Australia). These were termed SLN-A. These were then complexed with plasmid DNA encoding the UreA subunit of *H. pylori.* The resultant particles were termed lipoplex-A and were used for vaccination. The purification of recombinant UreA from *Escherichia coli* was undertaken as described in Skakic et al. (2023) [27].

Particle size distribution measurements were performed with DLS using the Malvern Zetasizer Nano ZS (Malvern Instruments, London, UK) and analysed using the Zetasizer Nano Software version 2.0. For size and zeta potential measurements, samples were measured at a concentration of 10 µg/mL in PBS in a DT1070 folded capillary cell and measured at 4 °C. Both SLN-A and lipoplex-A were stained and imaged using the JEOL1010 transmission electron microscope (JEOL, Tokyo, Japan) at 80 kV accelerating voltage at the RMIT Microscopy and Microanalysis Facility, RMIT University, Melbourne.

### 4.2. Immunisation of Mice

Six-week-old female C57BL/6 mice were purchased from the Animal Resource Centre (Canningvale, Western Australia) under approved animal experiment protocols (AEC1820). Fifty-six mice were randomly assigned to groups of eight mice. Mice were immunised either orally (Group A) or subcutaneously (Groups B-G) as per the vaccination regime outlined in Table 1. 

Briefly, the mice were vaccinated twice (except Group A), two weeks apart with either positive, negative, or internal control vaccines, or the experimental lipoplex-A vaccine with either lipoplex-A or recombinant protein boost. Positive control mice were vaccinated with a single oral dose of 1 × 10^7^ CFU attenuated *Salmonella enterica* serovar Typhimurium (*S. typhimurium* SL3261pYZ97) expressing UreA and UreB subunits [28]. Negative control groups B and C were sham vaccinated with PBS, and internal control group D received a lipoplex-A vaccine dose containing an empty plasmid vector (pcDNA3.1) to assess any non-specific immunogenicity of the carrier platform without the presence of the UreA gene. 

### 4.3. Challenge with H. pylori

*H. pylori* Sydney strain 1 (SS1) was grown in a broth culture in brain heart infusion broth (Oxoid, Scoresby, Australia), containing 10% foetal calf serum (FCS) (ThermoFisher, Scoresby, Australia) supplemented with vancomycin, trimethoprim, and nystatin, as previously described [29]. Twenty-eight days after the first vaccination, mice were challenged orally with 1 × 10^7^ CFU *H. pylori* strain SS1 via oral gavage with a stainless steel gavage needle. Mice in group C were not challenged and therefore served as a negative control for *H. pylori* infection. Twenty-one days after the *H. pylori* SS1 challenge, the mice were killed, and tissues and whole blood were collected for analysis.

### 4.4. Blood and Tissue Collection

To characterise antibody responses from vaccinations alone, blood was collected from the mice via saphenous vein bleed directly prior to challenge. To further characterise antibody responses after the oral *H. pylori* challenge, blood was collected from the mice at the termination of the experiment via cardiac puncture, three weeks after the oral *H. pylori* SS1 challenge. Serum was prepared from whole blood and stored at −20 °C until use. 

Stomachs were harvested immediately via dissection using sterile scalpels and forceps. Stomachs were removed and cut longitudinally for determination of the bacterial load or flow cytometric analysis. 

### 4.5. Antibody Determination by ELISA Assay

To determine specific titres of whole IgG and subtypes IgG1 and IgG2c, blood was collected from mice 3 weeks after vaccination, and again at the termination of the trial. ELISA assays were performed using recombinant UreA protein as described by Skakic et al. (2023) [27].

Briefly, antigen-specific IgG, IgG1, and IgG2c titres for individual mice were determined using indirect ELISA. Sera obtained from immunised mice were used as the source of the primary antibody, which bound to plates pre-coated with recombinant UreA. The titre cut-off point was determined based on the mean and three standard deviations from the negative control readings, and PBS naïve mouse titres were used as a baseline. Antibody levels were expressed as a logarithm of the titre. Statistical significance was determined via the Kruskal–Wallis test with Dunn’s Multiple Comparisons test and significance is represented as * *p* ≤ 0.05, ** *p* ≤ 0.01, *** *p* ≤ 0.001, **** *p* ≤ 0.0001.

### 4.6. Immune Cell Population Quantification by FACS

Gastric lymphocytes were extracted from gastric tissue as described by Skakic et al., (2023) [27], and the cell suspension counted and adjusted to a concentration of 10^6^ cells/mL prior to staining. Cells were prepared for antibody staining as described in Skakic et al. (2023) [27]. The following antibodies were used for surface staining: CD3 (145-2C11), CD45 (30-F11), CD4 (GK1.5), CD8 (53.6.7), CD11b (M1/70), Ly6G (1A8), and IFNγ (XMG1.2) (all from BD Biosciences, Franklin Lakes, NJ, USA). Cell population data were collected using a BD Canto II flow cytometer (BD Biosciences, NJ, USA) and analysed using FlowJo software (Version 8.5, BD Biosciences, NJ, USA).

Cells were gated according to their forward scatter (FSC-H and FSC-A) characteristics to exclude aggregates, and then further differentiated into a leukocyte gate based on their FSC-A and side scatter (SSC)-A profiles. Lymphocytes were gated on CD3^+^ to differentiate T cells, which were further divided into CD4^+^ T cells or CD8^+^ T cells, as well as IFNγ^+^ populations of each T cell subset. Macrophages were defined as CD3^−^/CD11b^+^ and neutrophils as CD3^−^/CD11b^+^/Ly6G^+^.

### 4.7. DNA Extraction from Mouse Stomach Homogenates and H. pylori Burden Analysis via Quantitative PCR

Mouse stomach homogenates were centrifuged at 14,000× *g* for 1 min and supernatant was discarded. DNA was extracted as described by Skakic et al. (2023) [27]. Stomach DNA samples were analysed via qPCR to detect the presence of *H. pylori* 16S rDNA and quantify burdens against a set of known *H. pylori* standards ranging from 0.0001–100 ng, as previously described [57]. Samples were amplified in 20 µL PCR reactions, containing an estimated 100 ng DNA, 1 µL of each forward and reverse primer (400 nM, Table 2), and 10 µL SYBR™ No-ROX Master Mix (Meridian Bioscience, Lukenwalde, Germany), made up to a final volume of 20 µL with nuclease free water. Samples were run on a Qiagen Rotor-Gene Q Platform version 2.3.4 (Qiagen, Germantown, MD, USA) using the standard manufacturer software. Samples underwent amplification for 30 cycles with a melting temperature of 56 °C, using group C (naïve control) to set the baseline, above which a signal threshold was applied. Bacterial counts per gram of stomach tissue were determined via extrapolation from the standard curve of known *H. pylori* DNA concentrations. 

## Figures and Tables

**Table 1:** Experimental groups for vaccination.

Group	Vaccine	Boost	Dose per Vaccination	Challenged
**A**	*S. typhimurium* SL3261pYZ97	-	1 × 10^7^ CFU	✓
**B**	PBS-sham	PBS	100 µL PBS	✓
**C**	PBS-sham	PBS	100 µL PBS	✗
**D**	Lipoplex-A (empty plasmid vector)	Lipoplex-A (empty plasmid vector)	50 µg DNA + 100 µg SLN-A	✓
**E**	DNA only (pcDNA-UreA)	DNA only (pcDNA-UreA)	50 µg DNA	✓
**F**	Lipoplex-A	UreA protein	50 µg DNA + 100 µg SLN-A, 50 µg UreA protein (boost)	✓
**G**	Lipoplex-A	Lipoplex-A	50 µg DNA + 100 µg SLN-A	✓

**Table 2:** *H. pylori* 16S primers for qPCR amplification [58].

FORWARD	5′-CTTAACCATAGAACTGCATTTGAAACTAC-3′
REVERSE	5′-GGTCGCCTTCGCAATGAGTA-3′

## Data Availability

Data is contained within the article.
